# Albuminuria in Pregnancy
*Paper opening a discussion at at meeting of the Bath, Bristol and Somerset Branch of the British Medical Association held on 25th May, 1932.


**Published:** 1932

**Authors:** R. S. S. Statham

**Affiliations:** Professor of Obstetrics, University of Bristol; Hon. Obstetrician, Royal Infirmary, Bristol


### ALBUMINURIA IN PREGNANCY.*

BY

R. S. S. STATHAM, M.D., Ch.M.,

*Professor of Obstetrics, University of Bristol; Hon. Obstetrician, Royal Infirmary, Bristol.*

---

THE object of this paper is to endeavour to assess the significance of the presence of albumin in the urine of a pregnant woman. It is obviously of the greatest importance to both midwife and doctor that a condition which is rapidly tending to a catastrophe should be recognized and promptly dealt with, while it is equally important not to frighten the patient and her relatives by needless excitement and alarming prognosis.

For clinical purposes it is convenient to regard the presence of albuminuria with pregnancy under five headings:—

1. Cases in which it is of no pathological significance.
2. Cases of infection of the urinary tract.
3. Cases of previous nephritis associated with pregnancy.
4. Cases of so-called "pregnancy toxæmias."
5. Low reserve kidney.

* Paper opening a discussion at a meeting of the Bath, Bristol and Somerset Branch of the British Medical Association held on 25th May, 1932.

The association of albuminuria with pregnancy is surprisingly common. In the six years between 1926 and 1931 793 cases were investigated at the Bristol Royal Infirmary. During this time about 8,000 patients attended the ante-natal clinic, so that albuminuria — to an extent which necessitated investigation—was noticed in 10 per cent. of all pregnancies.

Out of these 793 cases 81 were due to pyelitis, 50 were due to eclampsia with fits, 21 were due to toxic vomiting, 1 was due to acute yellow atrophy, 21 were due to acute nephritis ; making a total of 174 cases.

There were eleven deaths from eclampsia out of fifty cases, but of these nine were admitted very ill indeed from outside. During the same time only eight cases with one death developed on our own District. I have had difficulty in analysing the remaining 619 cases, but roughly one-half were apparently not pathological, and 300 were very mild nephritis, or "low reserve kidney," or mild toxæmic conditions including pre-eclampsia, *i.e.* about 60 per cent. of mild albuminurias were pathological to some degree.

To return to the five groups.

1. In a number of women traces of albumin appear in the urine for no apparent pathological reason. It is, of course, necessary to obtain a catheter specimen to avoid contamination from vaginal and vulvar secretions. The condition is commonly associated with large pregnancies, twins and hydramnios, and there may be some œdema of the feet also. These cases should be carefully investigated before deciding that the albuminuria is of no significance, and the condition calls for careful observation during the pregnancy, although it seldom produces any real pathological symptoms.

It is of interest to note here that there are definite but slight changes in the urinary tract during every normal pregnancy. These consist of slight changes in the renal parenchyma, which have been dignified by the name of " pregnancy kidney " by some German authors. There is also a well-developed hypertrophy of the lower end of the ureters (Hofbauer[1]), and Baird[2] describes a very definite dilatation of the ureters in which the right side predominates. These conditions probably determine a number of cases in the next group.

2. *Cystitis* will, of course, produce an albuminuria. It is not very common, and when present is an indication to suspect a gonococcal infection, unless the patient is a multipara with chronic cervicitis, which is known not to be gonorrhœal.

*Pyelitis* is very much commoner than is suspected. Dodds[3] found bacilluria due to B. coli in 6 per cent. of pregnancies, and it varies very much in the intensity of its attack. Treatment is usually successful in a short space of time. In the milder type of case equally good results are obtained from alkalinization of the urine, or from intra-vesical mercurochrome. The pain and temperature subside rapidly, and it is not usual, though not very infrequent, for the attack to recur. In severe cases there is nothing to equal an indwelling ureteric catheter of large size. The effects are dramatic in cases where there is pyonephrosis as well as pyelitis. There need be no fear of leaving the catheter in for several days. It should be gently syringed daily with 1 per cent. mercurochrome. Any case which does not respond to treatment in a reasonably short time should be X-rayed, as it is highly probable that a renal calculus will be discovered. This is so much the case that I consider failure to respond to treatment to be almost diagnostic of stone. Out of forty-two cases treated at the Royal Infirmary before 1926 there were two deaths, and in each case there was a large calculus and pyonephrosis. These two cases were early in the series and were not detected, as we had not realized the necessity for X-rays in cases which resist treatment, and they would almost certainly have been saved at the present time. Between 1926 and 1932 we treated eighty-one cases with no death and no induction of labour. With this line of treatment it should never be necessary to terminate a pregnancy for pyelitis, though the febrile and toxic condition of the mother occasionally causes death of the fœtus and abortion. The condition, unfortunately, tends to recur at subsequent pregnancies, and some permanent damage is done to kidney and ureter (Williams[4]).

3. *Cases of previous nephritis associated with pregnancy.*—This next group is the first of the "real albuminurias." They amount to about 30–35 per cent. of the cases. In many of the cases a history of previous nephritis, or of diseases such as scarlet fever, can be obtained. The prognosis as to the pregnancy is not good, since the association between abortion, or accidental hæmorrhage, and nephritis is so close. The prognosis for the patient is also none too good, even when the pregnancy is early. The renal condition frequently tends to become exacerbated about the third or fourth month, and placental infarcts occur which may interfere with the fœtal development or cause abortion. In exacerbation the diagnosis is usually simple, for the history of previous nephritis is commonly volunteered by the patient. General weakness, headache, and mild visual disturbances are usually the first symptoms, and an increasing œdema is an early sign. The urine is frequently copious in quantity and of low specific gravity, with a moderate degree of albumin present.

Treatment consists in rest in bed, appropriate diet, and medical treatment, combined with careful observation for any signs of rapid deterioration. I attach much importance to the appearance of albuminuric retinitis, and also to a sudden and rapid decrease in the daily amount of urine excreted. These and other signs of approaching uræmia are an absolute indication for termination of the pregnancy. It is my very strong opinion that it is not the province of the obstetrician to make the final decision in these cases, but that the advice of an experienced physician should be obtained in all cases where the pregnant patient is known to have a pre-existing nephritis. To my mind the case should be regarded as one of nephritis complicated by pregnancy, and should therefore be under the care of an expert in kidney disease, with the obstetrician in the background to deal with obstetric complications. This procedure is carried out at our ante-natal clinic at the Bristol Royal Infirmary with most satisfactory results, all cases of nephritis being put under the care of one of the physicians, who decides as to whether the pregnancy should proceed or be terminated, and keeps in touch with our department. Stieglitz[5] followed up fifty-three cases, and found that 80 per cent. were in a much worse condition three years after confinement, and each pregnancy increased the renal damage and arteriosclerotic changes.

4. *Pregnancy toxæmias.*—I include here the whole group of cases in which pregnant women exhibit "toxic" signs accompanied by albuminuria. These signs may point to a diagnosis of *true toxic pernicious vomiting, acute yellow atrophy of the liver, pre-eclampsia,* or *eclamptic* convulsions. Personally, I hold the view that these are protean manifestations of a pregnancy intoxication which affects all the organs in the body, with an especial predilection for the liver, kidney and brain.

This is not the occasion for a discussion on the pathology of these conditions, but I wish to point out that the liver is *always* affected, areas of hæmorrhages and focal necrosis being invariably present, and usually associated with albuminuria and a raised blood-pressure. In consequence of this generalized toxæmia the differential diagnosis from nephritis and pregnancy is not so difficult as it would appear at first sight.

A simple clinical test for diacetic acid and acetone (if positive) will strongly predispose to a diagnosis of pre-eclampsia, and is a routine in our ante-natal clinic in all cases of albuminuria. In pre-eclampsia the symptoms are very similar to those of pregnancy associated with severe nephritis: headache, nausea, giddy attacks, spots before the eyes are common at the onset. Œdema of limbs and face is usually observed. If untreated there is almost always some degree of mental confusion and sometimes sudden loss of vision.

At the Infirmary we are accustomed to lay stress on two laboratory findings to clinch the diagnosis. First, the reading of the diastase reaction is always very high in toxic cases, sometimes well over 150, while it is as low as 20–30 units in most cases of chronic nephritis. Also, the ratio of the nitrogen excreted as ammonia to that excreted as urea is raised in toxæmia and not in incipient uræmia. I regard an ammonia-nitrogen excretion of 20 per cent. as a precursor of eclamptic convulsions.

It follows, then, that any case of albuminuria especially associated with the signs and symptoms detailed above should be at once investigated upon these lines. If the results suggest nephritis the patient should be referred to a physician for observation and treatment. If the results confirm a diagnosis of pre-eclampsia the case is primarily an obstetric matter and is treated as follows: Absolute rest in bed is essential. No food of any description is allowed by the mouth, except water and glucose, which may be given *ad libitum* or till glycosuria appears. (I have found that most pre-eclamptics will take 10–14 oz. daily of glucose without eliminating it in the urine.) Purgation by salines is carried out daily, and the blood-pressure taken and urine tested not only for albuminuria, but for ketones, and an estimation made of the ammonia-urea ratio. In most cases there is an immediate and rapid improvement, the œdema decreases, the visual and mental disturbances lessen, and the urine increases in quantity, while the amount of albumin lessens and the blood-pressure falls. The patient is now allowed a mild carbohydrate diet. We usually begin with rusks, toast and honey or jam, and proceed to rice and potatoes. No fat or protein is allowed beyond the minimal quantities in the above dietary.

It cannot be too strongly stressed that milk is a most improper diet in these cases. It is seldom realized that one pint of milk contains almost an ounce of fat and also of protein, and very few would put a pre-eclamptic patient on a diet of an ounce of butter and the same amount of steak, yet a milk diet which far exceeds these quantities is very frequently ordered with disastrous results. The daily urine test and blood-pressure readings will show how the patient's liver is standing the increasing dietary, and in favourable cases, where food is gradually added, we prefer to allow proteins before fats. In an average case the patient is on a reasonably full diet in eight to ten days, and is then kept on a "low fat" diet, *i.e.* she is encouraged to take jam or syrup in preference to butter, and her milk is kept low, being given in tea and coffee, and not drunk neat.

In a certain number of cases the symptoms persist or even get worse, in spite of treatment. The blood-pressure rises and headache gets worse. If sudden blindness occurs it is of grave significance. These cases require immediate induction of labour, and I prefer the method of rupturing the membranes by a catheter. If the patient is in labour full "Rotunda"

or "Strogonoff" treatment should be instituted at once.

As this paper is intended to open a discussion upon albuminuria the detailed treatment of eclamptic convulsions lies outside its scope, and for the same reason the treatment of toxic vomiting and acute yellow atrophy is also omitted, as these are definite clinical entities of a nature which is quite obvious and more important than the mere symptomatic albuminuria.

5. The last group of cases comprises a rather mysterious clinical entity known as "low reserve kidney." I propose to leave this, in the greater part, to my medical colleague. I would draw attention to the fact that Whitridge Williams classified 35 per cent. of his albuminuria cases in this group.

The symptoms are general malaise and mild headache. The œdema is slight, if present at all, and the patient does not feel or seem really ill. The urine is fairly copious and the albumin small in amount. The blood-pressure is always raised, but rarely exceeds 160/90. As far as I can gather this group includes the cases which one cannot diagnose as definitely nephritic or pre-eclamptic, and so is a convenient label. Williams states that rest in bed and reduced diet cures practically all cases in one or two weeks, and states that permanent renal damage is rare. He regards these conditions as a mild toxæmia. Gibbens[6] classifies these as 10 per cent. obvious nephritis, and 50 per cent. occult nephritis, which only shows itself during pregnancy; while a recent paper by Browne[7] shows that chronic renal damage may exist between pregnancies without giving any signs at all, and he considers that this accounts for many cases described as "low reserve kidney."

REFERENCES.

[1] *Jour. Urol.*, 1928, xx. 413.
[2] *Jour. Obstet. and Gyn.*, 1931, xxxviii. 516.
[3] *Ibid*, 1931, xxxviii. 773.
[4] *Obstetrics*, 1927, p. 605.
[5] *Amer. Jour. Obstet. and Gyn.*, 1931, xxi. 26.
[6] *Lancet*, 1931, ii. 12.
[7] *Jour. Obstet. and Gyn.*, 1930, xxxvii. 476–482.

